# Ex Situ Breeding and Conservation of *Osmoderma* Species: A Systematic Review and Evidence-Based Breeding Guidelines for Reintroduction

**DOI:** 10.3390/insects17010094

**Published:** 2026-01-14

**Authors:** Šarūnas Kulbokas, Aurelija Mikalčiūtė, Gintarė Stankevičė

**Affiliations:** 1 Department of Research, Lithuanian Zoological Gardens, Radvilėnų Str. 21, LT-50299 Kaunas, Lithuania; aurelija.mikalciute@zoosodas.lt (A.M.); gintare.stankevice@zoosodas.lt (G.S.); 2 Department of Biology, Faculty of Natural Sciences, Vytautas Magnus University, Universiteto Str. 10, LT-53361 Akademija, Lithuania

**Keywords:** *Osmoderma*, breeding, ex situ, habitat requirements, conservation management, PRISMA

## Abstract

Hermit beetles (*Osmoderma* spp.) are protected and endangered across Europe, experiencing ongoing decline throughout most of their range. Because nearly all populations are small and isolated, *Osmoderma* genus is highly susceptible to extinction and requires active conservation measures. The primary cause of decline in the genus is habitat loss, particularly the removal of hollow trees that provide essential larval habitat. The nutritional wood mold within these hollows, on which larvae depend for 3–4 years of development, is directly linked to population survival. The aim of this study was to develop methodical ex situ breeding guidelines for reintroduction designed to eliminate environmental constraints and ecological requirement gaps. We first synthesize literature-based evidence on habitat conditions, applied methods, study durations, and key ecological insights relevant to *Osmoderma* conservation. Based on these results, we then create an ex situ breeding guideline for reintroduction, combining published data with practical breeding objectives in cases where empirical data are limited.

## Simple Summary

The unique hollow habitats that rare beetles depend on for survival are vanishing along with many of Europe’s veteran trees. Hermit beetles are one group that is in decline due to their slow growth, limited dispersal, and dependence on particular kinds of decaying wood. There is practically no helpful data on how to breed these beetles in captivity, which could assist in restoring decreased populations. In order to provide precise, useful guidelines for rearing and releasing them, we examined all of the research on their ecology, behavior, and breeding initiatives. We discovered that these beetles need extremely stable conditions: a rich mixture of decaying wood and leaves, warm, well-insulated tree hollows, and active microbial ecosystems that support larval growth. Young larvae require the proper substrate for several years and adults must be carefully captured during the short summer flight period. Additionally, we demonstrate how artificial nest boxes may effectively supplement natural hollows and promote complete development.

## 1. Introduction

Globally, most forests are being cut down at an enormous rate for various reasons [1]. The loss of old-growth forests, however, poses the biggest threat, as they offer unique continuity of deadwood habitats that support a variety of dependent organisms, as well as precious hollows with stable microclimates [2]. The IUCN [3] has classified many of these organisms as endangered or near threatened, with saproxylic beetles being one of the most at risk [4,5]. These beetles play a vital role in nutrient cycling, wood decomposition, and maintaining soil health [6]. Moreover, they depend on rotten wood and its nutrient-rich materials as their main food source, and their entire life cycle is closely linked to the microhabitats it produces [7].

The genus *Osmoderma*, which belongs to the family of saproxylic beetles, is a notable example [8]. These beetles are typically found in areas with a lot of old trees, especially with decaying oak trees [9]. The IUCN [3] indicates that nearly every species described in *Osmoderma* genus is on the decline. Reasons include extremely low metamorphosis completion rates [10], prolonged larval development lasting up to four years [11], dependence on the quality and availability of tree hollows during larval stages [12], and severely limited adult dispersal capacity with only about 15% of individuals leaving their natal tree [13]. For direct conservation efforts and ex situ breeding initiatives, precise taxonomic identification is crucial because of these distribution limits and resulting population isolation [14,15].

Proper animal taxonomy is one of the most important foundations for establishing an effective and evidence-based breeding program ensuring suitable individuals’ selection for the process [16]. *Osmoderma barnabita* (*O. barnabita*), which is found in Northern, Baltic, Central, and parts of Eastern Europe, was mistakenly identified and associated with *Osmoderma eremita* (*O. eremita*) for a long time after the first articles were published [11,17,18]. Since *O. eremita* is the only species mentioned in the EU Habitats Directive and the Bern Convention, most of the information only refers to this species [11]. However, recent genetic studies [19] have confirmed that *O. barnabita* is a distinct species. Thus, without correct recognition of its environmental requirements, habitat management strategies risk failing every single species [11]. Therefore, region-specific monitoring programs are essential to assess the status of the species in each country and to ensure that conservation actions are based on exact data rather than outdated or misleading assumptions [20].

Ex situ programs are more common in plants [21] and vertebrates [22]. Though, ex situ conservation and breeding have become increasingly popular methods complementing in situ protection in recent years [23]. This is especially important when there are significant bottlenecks or habitat fragmentation affecting natural population [24]. Current studies encompassing endangered butterflies like *Maculinea* spp. and *Euphydryas editha* [25] have shown the viability and importance of ex situ breeding. These studies reveal the potential of raising insects in captivity, as well as its complexity, since for many species completing their life cycles, specific substrates, conditions, and microflora are crucial [26]. Preventing further decline of isolated populations requires systematic ex situ breeding guidelines for reintroduction of *Osmoderma* beetles.

The core aim of this review is to provide a structured basis for ex situ breeding for reintroduction. Aiming to identify environmental constraints and ecological requirements relevant to effective ex situ breeding and long-term conservation, we conducted a systematic literature review. Key limitations affecting *Osmoderma* populations were identified using the reviewed studies, and these conclusions were subsequently converted into practical management recommendations.

This article examines the principal constraints shaping *Osmoderma* conservation, including restricted dispersal, dependence on hollow trees with suitable wood mold, sensitivity to temperature and humidity extremes, and susceptibility to demographic instability. Drawing on reviewed studies and practical breeding experience, we outline how these ecological requirements and limitations influence population dynamics, habitat use, and management decisions, thereby providing a structured basis for developing effective ex situ breeding with reintroduction programs.

## 2. Materials and Methods

A lack of specific research on *Osmoderma* species caused the choice to conduct the systematic review. This review was carried out between 14 July and 20 July 2025, following the PRISMA model to ensure transparency, reproducibility, and rigor in data collection and synthesis [27].

### 2.1. Information Sources and Search Strategy

Seeking to capture data relevant to breeding and the larval development of *Osmoderma* species, primary articles were searched via 7 major scientific databases (e.g., MDPI, ResearchGate, SpringerLink, Wiley, Taylor & Francis, Elsevier, and ScienceDirect) using the following keywords: (“*Osmoderma*” AND “larval development” OR “breeding” OR “rearing”). Due to limitations in search functionality on MDPI and ResearchGate, only “*Osmoderma*” keyword was used in these platforms. Despite the fact that *Osmoderma* genus includes saproxylic beetles, the keyword “saproxylic beetle” was rejected due to an overly broad range of results, many of which would be unrelated to *Osmoderma* species. The initial search found 399 records.

### 2.2. Inclusion and Exclusion Criteria

Inclusion criteria (IC):

IC1. Written in English

IC2. Explicit focus on *Osmoderma* (title, abstract, or keywords)

IC3. Contains data on breeding, rearing, larval development, or ex situ conservation

IC4. Full text accessible

Exclusion criteria (EC):

EC1. Not about *Osmoderma*

EC2. Not relevant to breeding/larvae/ex situ topics

EC3. Not written in English

EC4. Full text unavailable

EC5. Duplicate

**Figure 1:**
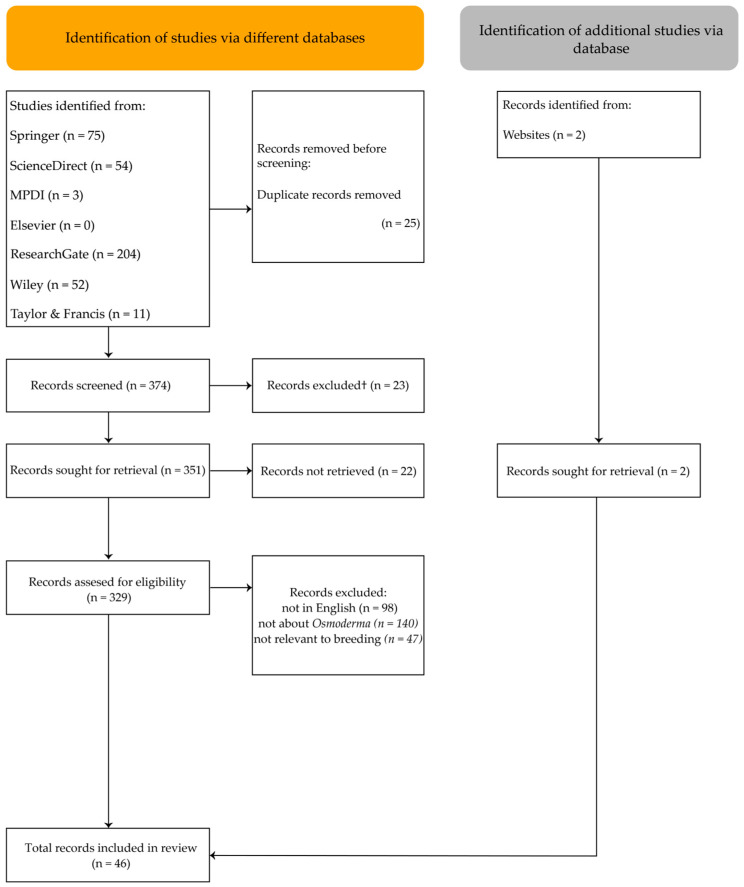
PRISMA flow diagram.

After removing 25 duplicate entries, 374 records remained for screening. Of these, 23 records were excluded for not meeting eligibility criteria, as the search found 1 incomplete article with only an abstract, and 22 records could not be retrieved in full. The remaining articles were assessed for inclusion based on the following criteria: (1) written in English; (2) the main focus or keywords/title/abstract specifically addressed *Osmoderma*; and (3) relevance to breeding, rearing, larval development, or ex situ release (Figure 1).

### 2.3. Study Selection Process

The screening was performed by two assessors in parallel to practice the application of the inclusion and exclusion criteria. After this step, assessors discussed results and refined the established criteria. Following this session, the assessors independently and blindly evaluated each record’s title and abstract. Disagreements were discussed. After the selection of papers for full-text analysis was complete, the papers were retrieved. The two assessors read the full texts using the same eligibility criteria. Both worked in parallel and were blind to each other’s choices. Moreover, we had to agree on the exclusion of records. No automation tools were used for screening or data extraction. The review protocol was not registered.

After screening, 44 articles met all criteria. However, none provided information on active ex situ breeding programs. Reaching the aim of this review, additional searches were conducted in official LIFE project database for *Osmoderma* beetles breeding; therefore, 2 additional papers were included in the final review.

### 2.4. Data Extraction and Synthesis

Each eligible study’s data was extracted and put into a standardized spreadsheet (Table S1). The year of study, geographical region, research focus, study type, and key findings relevant to ex situ conservation were among the variables that were extracted. To find reoccurring patterns and knowledge gaps across studies, data were narratively analyzed with the help of summary tables.

### 2.5. Methodological Limitations

It is important to acknowledge the methodological limitations of this systematic review. Due to the exploratory focus of this synthesis in a field with a limited and strongly dispersed amount of literature, the review process was not pre-registered (e.g., PROSPERO or OSF). Furthermore, since most eligible studies were descriptive, observational, or methodological types, which limited the use of standard bias assessment methods, no formal quality or risk-of-bias evaluation was carried out. Finally, “ResearchGate” was added as an information source to capture publications related to *Osmoderma* biology and breeding that would otherwise be unavailable or difficult to retrieve, in addition to major publisher databases.

## 3. Results and Discussion

**Figure 2:**
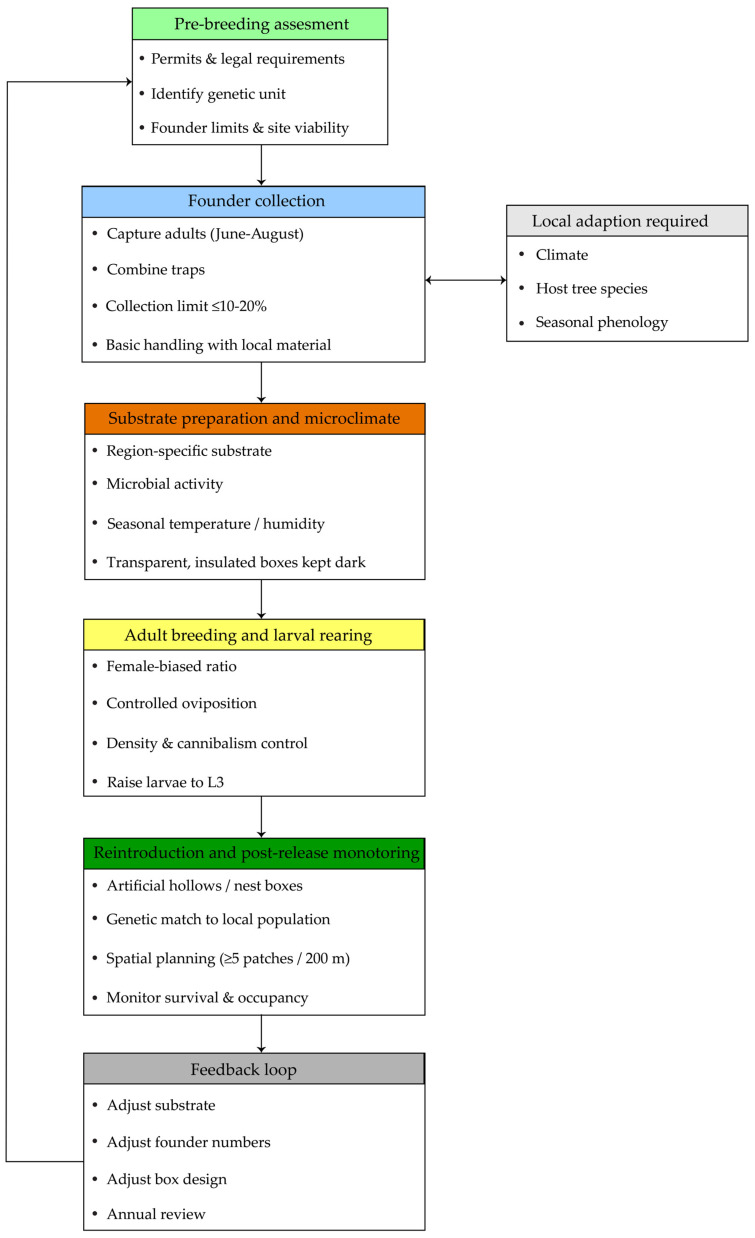
Evidence-based ex situ breeding guideline for *Osmoderma*.

Based on the findings of the systematic review, we summarized the ecological and biological requirements consistently supported by the literature. An ex situ breeding guideline for reintroduction was then developed by integrating the results of the systematic review with researcher knowledge and practical breeding experience, with literature-supported findings presented separately from expert-informed recommendations (Figure 2).

### 3.1. Ecological and Practical Constraints Shaping Osmoderma Breeding

The *Osmoderma* beetles serve as valuable umbrella species and indicators of forest ecosystem integrity [10,28]. Despite growing research, the conservation and ex situ breeding of this species faces challenges mostly related to ecological factors and habitat conditions [20,29,30].

The practical significance of conserving *Osmoderma* species extends beyond their survival, as it contributes to maintaining ecosystem functions and biodiversity [31]. In this review, we integrate the results of the systematic literature synthesis into practical ex situ breeding recommendations that are primarily applicable to populations of *Osmoderma barnabita* in Northern and Eastern Europe (e.g., Lithuania, Latvia, Poland), reflecting the geographic distribution of the empirical research that is currently available. We also go over the adjustments needed for *O. eremita* populations in Central and Southern Europe where there is adequate data, especially in relation to variations in phenology and substrate composition. The applicability of these recommendations outside of the researched areas should be carefully assessed and tailored to local biological and climatic conditions due to the uneven distribution of evidence between locations. The following sections present a structured conceptual overview based on locally accessible data to assist practitioners in organizing, carrying out, and assessing ex situ breeding for reintroduction.

Most reviewed studies were observational, field-based and employed different research for assessing *Osmoderma* ecology and population status, such as classic and pheromone-baited traps, headspace analysis, mark–release–recapture, mtDNA COI sequencing, radio telemetry, and gas chromatography–mass spectrometry [18,19,32,33,34]. Study duration mostly included seasonally for 1 or 2 years, but this term is not always right, especially when population structure is based on Monte Carlo simulation [12,20,33]. Most studies present results linked to Sweden, Italy, and France [8,20,31,34,35,36,37]. However, different geographical areas need specific research, especially in overlap zones where sympatric species occur and must be managed as separate units [19,38]. Though reviewed studies encompass *Osmoderma* species, research focus includes examining habitat requirements [12], investigating population structure and dynamics [34,39], addressing larval diet [40,41], examining distribution ability [42], and exploring pheromone and monitoring approaches [13,43]. Therefore, only several studies [44,45] directly present implications for breeding programs. Strong emphasis on physical habitat requirements and population dynamics of *Osmoderma* species serves as the conceptual approach for an effective ex situ breeding program preparation.

### 3.2. Physical Habitat Requirements

The hermit beetle occupies veteran cavities formed over years in broad-leaved trees [8]. In most countries, old oaks (*Quercus robur*) dominate as host tree. Despite that, research shows that lime (*Tilia*), beech (*Fagus*), ash (*Fraxinus*), willow (*Salix*), hornbeam (*Carpinus*), and alder (*Alnus*) trees can also support populations [10,11]. Occupied hollows are typically located 1–6 m above ground [9]. Moreover, hollow entrances are often exposed to sunlight, commonly south- or west-facing [30]. Occupancy increases sharply with cavity volume and wood mold quantity. Small quantities of wood mold often indicate no occupancy, and tree quality nearly fully determines tree occupancy patterns [12]. Furthermore, the continuous removal of tree cavities by forestry practices, such as removing hollow trees or turning stands to coppice, accelerates habitat loss, and juvenile pollards frequently lack adequate hollows [20]. These risks are further exacerbated by anthropogenic impacts. The landscape has changed dramatically as a result of early tree cutting, urbanization, ecological fragmentation, and the conversion of natural stands to coppice [19].

### 3.3. Population Structure and Dynamics

The long-term survival of *Osmoderma* species in the wild is threatened by a combination of ecological constraints and human-induced pressures. Beetles’ low dispersal ability (only 15% of individuals attempt to disperse, and movements usually extend less than 200 m) is one of their most significant challenges [13,32,34,46,47]. As a result, populations often remain limited to single trees, with males showing extreme site fidelity, and gene flow between hollow tree habitats is severely restricted [39]. In fragmented environments, this restricted mobility leads to a distinctive metapopulation structure [48,49], in which isolated populations can survive for several generations before eventually collapsing [19,50].

Nevertheless, extinction risks are greatly increased by small populations [51]. Sex ratios tend to be male-biased, ranging from 0.78 to 6.9 [35], and seasonal numbers per tree rarely exceed 100 individuals, increasing the possibility of reproductive collapse [50]. *Osmoderma* populations are particularly vulnerable to biological stochasticity due to their low densities, significant fluctuations, and density reliance when compared to the majority of other saproxylic insects [12].

Additional pressure is added by environmental factors. The species is prone to human-caused stresses, temperature thresholds, and climatically driven occurrences [52]. Those combined factors increase the probability of local extinction and long-term decline when coupled with habitat loss and fragmentation, which restrict dispersal and isolate populations [13,51,53].

Thus, targeted conservation measures are crucial in considering these limitations. To prevent population fragmentation and ensure the survival of the species, active management interventions and ex situ breeding efforts may be more important than habitat protection alone.

### 3.4. Guidelines for the Effective Ex Situ Breeding of Osmoderma Species

Based on the reviewed studies, we propose a practical four-step structure for ex situ breeding and reintroduction that is primarily informed by case studies from Northern and Eastern Europe. This structure includes: (1) collection of founder adults and larvae from the wild, (2) preparation of species- and region-specific substrates, (3) adult breeding and larval rearing under controlled conditions, and (4) reintroduction using artificial hollows or nest boxes. For each step, we describe the recommended timing, target life stages, and applied methods, while explicitly indicating aspects that require regional or population-specific adaptation, such as climate, host tree species, and the taxonomic identity of local *Osmoderma* populations.

#### 3.4.1. Collection of Adults and Larvae from the Wild

A successful start requires the capture of founder individuals from the wild only for conservation and only with right permits. At present, no standardized, quantitative criteria exist for selecting optimal founder individuals in *Osmoderma*, and founder choice is therefore constrained primarily by legal protection status, availability, and ethical considerations. *Osmoderma* species are strictly protected under national legislation and the EU Habitats Directive [11]. Any removal of individuals must be authorized within an approved conservation plan [44,45]. Given that, founder collecting is mostly limited to adult stage, its timing varies by the species’ breeding period [11,52,54].

The adults have a two-month lifespan and are actively seeking mates, while males use pheromones to attract females [34]. The best time to catch beetles is when they emerge as adults to reproduce, typically from June to August. This period can vary if temperatures are higher than average [52]. Table 1 outlines the optimum capture time, suggested techniques, and handling guidelines for *O. barnabita* and *O. eremita* as recommendations for practitioners.

Research emphasizes collection by hand and trapping as core methods to collect adults from the wild [8,20,34,56,57]. Different traps can be used for collecting beetles: pitfall traps (PTs) [13], black cross window traps (BCWTs), black bottle traps (BBTs) [54], passive pheromone traps (PPTs) [53] and smart pheromone traps (SPTs) [59]. PTs are gravity-based traps positioned directly in accessible hollows [13]. They are most effective for capturing both males and females present in cavity yet if the cavity is too small trap could not be hold securely [53]. BCWTs are cross-shaped transparent panels that capture flying beetles, combined with a lure holding synthetic (R)-(+)-γ-decalactone to attract males. Best succession is achieved when placed near the hollow, which is effective in detecting beetles’ presence of nearby metapopulations. BBT variant of the flight-capture trap design often has better structural design or location, particularly on trees with a lot of microhabitats [54].

The location of the trap and local factors may affect its effectiveness [42]. PPTs are non-mechanical hanging lures that draw beetles by releasing synthetic male pheromone. Although wind and habitat structure may restrict their area of attraction, they are easy to keep, require minimal maintenance, and helpful for monitoring [53]. SPTs are modern automated devices equipped with sensors, data loggers, or remote communication systems to record capture events without manual checks. While they reduce labor and allow continuous monitoring, studies have shown they often catch fewer beetles than conventional pheromone-baited traps [59]. Collecting individuals by hand is the method mostly used for collecting adult beetles from tree hollow entrances, cavity surfaces, or trunks [45,56]. This method is highly selective and allows researchers to target specific individuals, yet it requires skilled observers and a lot of time.

PTs baited with (R)-(+)-γ-decalactone caught 727 individuals as opposed to just 73 captured ones with smart traps [59]. Thus, newer technology-based traps are still not a reliable substitute for extensive monitoring. Although BBTs have recorded significantly more individuals (21 vs. 15), particularly on trees with numerous microhabitats, BCWTs and BBTs perform similarly in urban settings [54]. Since the pheromone attracts both females and males, pheromone-based methods are useful for identifying both sexes. However, the use of traps eliminates an opportunity to identify females directly, and their accuracy decreases as the compound deteriorates [42,53]. Furthermore, research carried out in North America and compared to European results have shown that pheromone-baited traps may be more effective at attracting females, while pitfall traps, which are positioned immediately inside tree hollows, may be more effective at attracting males [60].

Hence, the best method for capturing beetles for population sampling has proven to be traps [13]. Traditional traps often capture more individuals than their technologically advanced alternatives [59], particularly when baited with the synthetic male pheromone (R)-(+)-γ-decalactone [33]. In addition to adult trapping, trained conservation dogs can also detect larvae, which has been shown to be much more effective compared to other methods [38,56]. Vacuum suction method, on the other hand, runs the danger of harming specimens and is still not as suitable for conservation [58].

Although methods such as trapping with a PT, BCWT, BBT, PPT, SPT, collected by hand and trained detection dogs support collection results, traditional pheromone-baited pitfall traps remain more effective in catching *O. eremita*. While no single way guarantees thorough capture of this beetle, the most accurate sample options are provided by a combination of methods. Despite the technique of capture, caught specimens must be moved within a few hours into containers that have been filled with the breeding substrate mentioned in Section 2.

#### 3.4.2. Substrate Composition and Larval Diet

Replicating the microbial and physical characteristics of natural tree hollows is necessary to create breeding substrates that are effective for *Osmoderma* species [18]. Research [40,61] shows significant requirements for substrates as they depend on geographic region, meaning that management must be adapted to local conditions.

The highest larval survival rates for *O. barnabita* in Lithuania have been observed when locally sourced, brown-rotted oak wood (40–50%) is mixed with composted oak leaves (50–60%). It is also critical to change leaves annually, because they deteriorate and the larvae are left without supplementary food [45]. These proportions are based on applied rearing practice, as experimentally defined quantitative nutrients or structural thresholds for optimal larval survival are currently unknown.

The nutritional value of organic waste extends beyond wood, as studies show that although *O. barnabita* larvae primarily feed on decomposing oak material, they additionally consume leaf litter, frass, and compost-enhanced detritus like peat and animal manure [40]. Thus, it has been proposed that *O. eremita* can thrive on a mixed substrate that includes 50% beech sawdust, 25% animal manure, and 25% peat, which should be turned every six months to encourage microbial activity [44]. These results show how important it is to incorporate species- and region-specific adjustments into any guidelines for captive breeding or ex situ conservation. However, these substrate ratios are not based on quantified nutritional thresholds, which have not yet been established for *Osmoderma* larvae.

The biology of the species links with seasonal change since larval feeding activity only takes place in the summer, when daily temperatures reach over 13 °C [10,11]. To replicate natural overwintering behavior, temperature and humidity levels should be seasonally adjusted. Exact thermal and humidity thresholds for optimal larval development have not yet been defined [52].

Substrate’s humidity level is essential for larval growth and could be regulated by removing coarser wood pieces and regular irrigation [40]. Humidity and temperature parameters should be maintained throughout the life cycle of *Osmoderma* and seasonally adjusted for regular steady growth [20,30,52]. Quantitative moisture thresholds have not been experimentally determined, and current recommendations are based on observed larval performance rather than defined limits.

Growth is greatly affected by the larval microbiota, which includes yeasts of the *Debaryomyces*, *Schwanniomyces*, and *Candida* genera as well as nitrogen-fixing fermentation chambers [41]. These microbial colonies demonstrate that the quality of a substrate must be assessed based upon its physical characteristics and its ability to sustain microbial activity [40,41]. Larval growth rates are not only influenced by microbial biomass but also mineral content, especially phosphorus (P), potassium (K), sulphur (S), and nitrogen (N) [40]. Gut-associated microbes facilitate the breakdown and transformation of frass into edible material, making it a crucial food source in areas with limited resources [17,31]. Thus, reusing frass in rearing boxes can aid in creating a substrate that is biologically active and promotes larval development [45]. At present, no quantitative benchmarks exist for microbial composition or activity associated with optimal larval growth.

However, not each potential substrate is suitable for species biological requirements. The leaf humus by itself is not sufficient as a sole dietary source, even though it produces rapid growth and a high percentage of larvae reaching advanced stages [30]. More importantly, larvae that only consumed *Laetiporus sulphureus* (*L. sulphureus*) mycelium died 100% of the time in a matter of weeks, proving fungal tissue is indigestible and cannot support life. Rather, *L. sulphureus* makes an indirect contribution by acting as a cavity-forming and wood-decomposing agent that promotes the release of nutrients from other sources [40]. Table 2 summarizes the key larval requirements, substrate characteristics, and husbandry considerations necessary for successful rearing across different *Osmoderma* species.

Consequently, sterile or inadequate substrates significantly reduce the growth and survival of larvae. The effectiveness of breeding emphasizes the importance of ensuring a microbially active, biologically rich substrate for the larvae’s development and health.

#### 3.4.3. Adult Breeding and Larval Housing

Ensuring larval survival and successful reproduction, ex situ breeding of *Osmoderma* species depends on maintaining strong, genetically representative adults [19]. Only sufficiently large populations to resist removal should be collected from the wild [12], and breeding plans should be implemented to avoid inbreeding while preserving genetic diversity [62]. Depending on the species, breeding environment, and observed behavior, sex ratios should be carefully managed to optimize mating opportunities. Female-biased systems (3:1) are currently the only described option that gives optimal results [45].

Females usually lay 20–30 fragile eggs in deeper substrate layers [11], and this has been confirmed in controlled breeding containers for *O. barnabita* in Lithuania [45]. Eggs and larvae should be left undisturbed until seasonal inspections due to their vulnerability; they undergo a 14–20-day incubation period, growing from 2–3 mm to 4–5 mm and changing color from greyish white to greyish yellow [40]. Crucially, there are no apparent variations in the appearance of eggs between *O. eremita* and *O. barnabita* [11].

The humidity and temperature should be likewise those that inhabit hollow trees in the wild. Microclimatic preferences observed in the wild reveal that *Osmoderma* beetles avoided excessively moist cavities and were more common in warmer, more stable environments [30]. Thus, air conditioning and misting to imitate natural moisture changes while keeping an ambient temperature of +18 to +22 °C and 75 to 85% relative humidity is essential during breeding [45]. Considering beetles’ native home tree hollows replication, the breeding containers should be kept in the dark [13,44,45].

Larvae develop in three stages (L1, L2, L3) and finish their entire metamorphosis inside pupae chambers. A fully developed L3-stage larvae can grow up to 75 mm in length, but a newly hatched L1-stage larvae is only around 6 mm [45]. Depending on the timing of oviposition and the local microclimate, the larvae overwinter at either the first or second stage throughout the 3 instars, which last for 2 to 4 years, before pupating and becoming adults [11]. Aiming to identify the success of larval development, the breeding containers should be checked twice a year [44,45]. Therefore, the most important inspection is in spring when both larvae and beetles in pupae chambers eclose from hibernation.

Several ways could be used while checking larvae. One way is to keep containers untouched to hold hatchling larvae until they reach the L2 stage. Then it should be re-distributed (about 20 larvae per box) and raised to L3 and pupation over a period of 2 to 3 years [11,44] to lessen the likelihood of cannibalism [40]. The other way is to guarantee an even development, so larvae need to be sorted, usually with 30 to 50 individuals per box and check for size every spring [45]. However, the number of larvae in the box should be sufficient to match the amount of feed in the box [40]. In addition, the substrate in each box should be supplemented with leaves to ensure a continuous food supply for the larvae [45]. Once larvae reach the L3 stage, preparations should be made for their release into suitable habitats [11,45].

Careful replication of the natural microclimatic conditions and structural settings of tree hollows is necessary to maintain larval survival, development, and future reproductive potential. Successful ex situ breeding of *Osmoderma* species requires conditions that closely mimic the beetles’ native habitat, such as the use of artificial boxes [30,57].

#### 3.4.4. Reintroduction

Most empirical evidence on artificial nest boxes for *Osmoderma* originates from Central and Northern Europe, particularly Poland and surrounding countries. Since natural hollow trees are frequently in lack or insufficient for ex situ breeding with reintroduction of *Osmoderma* species [57], artificial nest boxes are being used in increasing numbers.

Recent experimental research reveals that *Osmoderma* can complete its life cycle in artificial nest boxes. In Poland, L2 and L3 *O. barnabita* larvae in 18 out of 20 boxes survived. Final inspection showed 111 healthy larvae and 28 pupal chambers, indicating successful pupation and at least one new generation produced entirely inside the boxes [30]. Microclimatic measurements showed that average daily temperatures in the boxes typically differed by ≤1–2 °C. Thus, well-designed boxes do not expose larvae to abnormal thermal regimes [30,57]. Artificial boxes filled with oak leaves and sawdust have been successfully colonized and used for breeding in Poland [30]. Research now explicitly recognizes artificial boxes as a tool to fill spatial and temporal gaps in suitable habitat [11].

By containing biologically active substrates, these boxes imitate the microbial and structure of tree cavities [11,30]. While dimensions and designs can vary, artificial nest boxes are generally adapted to balance ecological function with monitoring practicality. Larger nest boxes (e.g., 250 × 50 × 50 cm) can be applied to support breeding and rearing while also providing ecological value for other hollow-dwelling fauna such as bats and birds [45]. Smaller versions (e.g., 70 × 40 × 30 cm) can be used in reintroduction trials where ease of placement, monitoring access, or local tree structure influence design choices [44].

Larval survival depends strongly on microclimatic conditions. First, instar larvae are highly vulnerable to heat stress, with mortality occurring above ~40 °C, whereas overwintering larvae can withstand temperatures as low as −7.5 °C [52,63]. To protect against extreme summer temperatures and winter freezing, artificial nest box insulation and positioning are crucial. As only about 2.4% of adults migrate between trees [50], *Osmoderma* has a low dispersal capacity, making spatial planning even more important. At least 5 occupied patches within 200 m are necessary to sustain gene flow [12,32,51]. Balanced substrate (>10 L of wood mold) can increase colonization rates, gene flow, and larval occupancy to about 75% [64].

Another essential element for release is genetic integrity. *O. barnabita* has strong genetic structure, with distinct clades across Europe and a genetically vulnerable northern population that was likely shaped by post-glacial expansion [19,62]. Reintroduction needs to give priority to founders who share descent with the being introduced population to maintain local adaptation and evolutionary potential [57]. Incorporating habitat design, environmental control, layout, and genetic considerations to reintroduction strategies is essential for long-term species survival.

While these findings demonstrate that artificial nest boxes can successfully support *Osmoderma* development under temperate European conditions, their effectiveness elsewhere will depend on regional microclimate, tree species composition, and local population genetics, requiring site-specific validation before large-scale application.

## 4. Future Research Proposals

Future research priorities reflect clear gaps identified in the reviewed literature. The majority of the data that is now available is from Sweden, Italy, and Lithuania. Large portions of the *Osmoderma* distribution range, especially in Northern and Central Europe, are still under-represented. Considering this, an important part of the species’ distribution lacks evidence-based, concrete guidelines for founder selection and performance evaluation. For founder selection in particular, clarification is required because subjective descriptions like “vigorous males” are unreliable and would benefit from precise, quantifiable measures of individual quality. Furthermore, even if artificial nest boxes aid in the development of larvae, nothing is known about the long-term destiny of released individuals. This highlights the importance of post-release monitoring of occupancy and survival to confirm the success of reintroduction.

A further key research priority concerns the microbiome of breeding substrates. Although microbial activity in wood mold is directly related to larval survival, little is known about the composition and functions of related bacterial and fungal communities. Since these components have not been measured in previous research, the quantitative threshold values for phosphorus, potassium, sulfur, and nitrogen in *Osmoderma* breeding substrates are currently unknown. In addition to strengthening long-term conservation outcomes for *Osmoderma* species, comparative research concentrating on substrate microbiome composition and nutrient dynamics will significantly increase the reliability and dependability of the ex situ breeding guidelines suggested in this review.

## 5. Conclusions

This review synthesized empirical data on habitat requirements, population structure, substrate composition, captive breeding and reintroduction methods for *Osmoderma* species. The primary focus was on *O. barnabita* and *O. eremita*. Examined studies show that the hermit beetle restrained by (i) dependence on hollowed veteran trees with large volume of wood mold, (ii) low dispersal rate and metapopulation structure, and (iii) strict substrate requirements during larval stage. The four-step ex situ breeding guidelines suggested in this research are based on these three ecological limitations, which also explain why many populations are small, isolated, and demographically vulnerable.

First, data from population and habitat studies shows that founder collection cannot be opportunistic. The quantity of wood mold, cavity volume, and microclimate are closely related to occupancy and larval presence. Besides that, only few adult beetles disperse more than a few hundred meters. This indicates that only well-connected, multi-tree clusters with large wood-mold volumes should be selected as founders. The review of trapping methods suggests that the most reliable and least disruptive way to find adults is to use pheromone-baited pitfall and flight traps together with hand searching. The recommendation is that founder collecting in ex situ programs should be based on focused, pheromone-assisted monitoring within habitable metapopulation habitats rather than isolated trees is directly supported by our findings.

Second, the substrate and diet studies provide concrete formulations and parameters for captive rearing. The highest survival rates for *O. barnabita* in Lithuania were found in substrates that mainly consisted of locally gathered, brown-rotted oak wood along with composted oak leaves. Successful substrates are microbially rich and contain decayed hardwood and frass, which is a common pattern across regions. Rather than relying entirely on sterile or leaf-only material, our results confirm the clear recommendation of using substrate mixes tailored to the region and species based on local tree species and to actively maintain microbial and frass components.

Third, the literature shows that *Osmoderma* larvae require specialized housing conditions in captivity and cannot be managed using general beetle husbandry guidelines. Disturbance and density management are crucial due to a long larval stage, overwintering at L1 or L2, and low yearly reproduction. Case study shows that cannibalism is decreased and full development to L3 and pupation is supported by female-biased breeding groups in dark, secure, hollow-like containers, low to moderate larvae numbers per box, and regular redistribution. These results justify the practical recommendations: maintain breeding containers in dark, warm, moderately humid conditions that mirror natural hollows, sort larvae by size at defined intervals, and match larval numbers to available substrate volume and food.

Finally, the nest-box and reintroduction studies show solid evidence that *Osmoderma* can complete their life cycle in artificial nest boxes. Experiments show high larval survival, numerous pupal chambers and at least one full generation produced within boxes. In light of this, well-insulated boxes closely track natural cavity temperatures, supporting the use of nest boxes as more than just monitoring. However, metapopulation models and dispersal statistics show that box placement needs to be considered. These species have low movement rates and require several occupied hollows within roughly 100 m to maintain gene flow. These findings collectively confirm the claim that artificial nest boxes with biologically active wood mold are an appropriate, though spatially sensitive, method for both in situ habitat improvement and ex situ rearing.

## Figures and Tables

**Table 1:** Practical capture protocol for *Osmoderma* species used in ex situ breeding.

Component	*O. barnabita*	*O. eremita*	Universal Recommendations	Authors
Flight period/capture window	June–early August		Capture on warm, calm days (18–25 °C); avoid late-season captures to prevent sampling weakened individuals	[11,35,42,52,55]
Capture method	Pheromone-baited pitfall traps + black cross window traps near old trees		Use (R)-(+)-γ-decalactone as standard lure; check traps every 24 h	[8,20,56]
Founder selection	Preferred females + vigorous males; take max. 10–20% of local adult population		Sample from ≥5 occupied trees, when possible, to increase genetic diversity	[36,39,57,58]
Handling	Place individuals into ventilated containers with moist local wood mold		Transport within hours; avoid heat stress and desiccation	[44,45]

**Table 2:** Key parameters for larval rearing.

Component/Feature	*O. barnabita*	*O. eremita*	Authors
Primary wood material	40–50% brown-rotted oak wood (from natural hollows or decayed logs)	50% decomposed beech or oak sawdust	[8,29,30,61]
Supplementary plant material	50–60% composted oak leaves, sieved	-	[45]
Organic enrichments	5–10% reused larval frass to inoculate microbial community	25% well-composted animal manure (horse/cattle)	[31,40]
Additional substrate fraction	-	25% peat or similar organic detritus	[44]

## Data Availability

The original contributions presented in this study are included in the article/Supplementary Material. Further inquiries can be directed to the corresponding author.

## Supplementary Materials

The following supporting information can be downloaded at: https://www.mdpi.com/article/10.3390/insects17010094/s1, Table S1: Annotated Comparative Table of Osmoderma Genus Studies.
